# Identification and characterization of levulinyl-CoA synthetase from *Pseudomonas citronellolis*, which differs phylogenetically from LvaE of *Pseudomonas putida*

**DOI:** 10.1186/s13568-019-0853-y

**Published:** 2019-08-13

**Authors:** Hiroshi Habe, Hideaki Koike, Yuya Sato, Yosuke Iimura, Tomoyuki Hori, Manabu Kanno, Nobutada Kimura, Kohtaro Kirimura

**Affiliations:** 1 Environmental Management Research Institute, National Institute of Advanced Industrial Science and Technology (AIST), Tsukuba West, 16-1 Onogawa, Tsukuba, Ibaraki 305-8569 Japan; 2 Bioproduction Research Institute, AIST, Tsukuba Central 6-10, 1-1-1 Higashi, Tsukuba, Ibaraki 305-8566 Japan; 3 Department of Applied Chemistry, Faculty of Science and Engineering, Waseda University, Tokyo, 169-8555 Japan

**Keywords:** Lignocellulose, Levulinic acid, *Pseudomonas citronellolis*, Acyl-CoA synthetase, Levulinyl-CoA synthetase

## Abstract

Levulinic acid (LA) is a building block alternative to fermentable sugars derived from cellulosic biomass. Among LA catabolic processes in *Pseudomonas putida* KT2440, ligation of coenzyme A (CoA) to LA by levulinyl-CoA synthetase (LvaE) is known to be an initial enzymatic step in LA metabolism. To identify the genes involved in the first step of LA metabolism in *Pseudomonas citronellolis* LA18T, RNA-seq-based comparative transcriptome analysis was carried out for LA18T cells during growth on LA and pyruvic acid. The two most highly upregulated genes with LA exhibited amino acid sequence homologies to cation acetate symporter and 5-aminolevulinic acid dehydratase from *Pseudomonas* spp. Potential LA metabolic genes (*lva* genes) in LA18T that clustered with these two genes and were homologous to *lva* genes in KT2440 were identified, including *lvaE2* of LA18T, which exhibited 35% identity with *lvaE* of KT2440. Using *Escherichia coli* cells with the pCold™ expression system, LvaE2 was produced and investigated for its activity toward LA. High performance liquid chromatography analysis confirmed that crude extracts of *E*. *coli* cells expressing the *lvaE2* gene could convert LA to levulinyl-CoA in the presence of both HS-CoA and ATP. Phylogenetic analysis revealed that LvaE2 and LvaE formed a cluster with medium-chain fatty acid CoA synthetase, but they fell on different branches. Superimposition of LvaE2 and LvaE homology-based model structures suggested that LvaE2 had a larger tunnel for accepting fatty acid substrates than LvaE. These results indicate that LvaE2 is a novel levulinyl-CoA synthetase.

**Electronic supplementary material:**

The online version of this article (10.1186/s13568-019-0853-y) contains supplementary material, which is available to authorized users.

## Introduction

In recent years, much attention has been paid to the catalytic transformation of lignocellulose into key building block compounds under relatively mild conditions. An alternative building block to fermentable sugars is levulinic acid (LA), which is formed from cellulose or sugars in the presence of large quantities of mineral acids (Hayes et al. 2006; Pileidis and Titirici 2016) and is considered one of the top 12 building blocks by the US Department of Energy (Werpy and Petersen 2004). Although high concentrations of homogeneous acids such as H_2_SO_4_ and HCl have long been used for the synthesis of LA (Girisuta et al. 2008), heterogeneous acid catalysts have been applied recently to avoid the harsh and corrosive properties of mineral acids and the difficult recovery of such acids (Chen et al. 2011; Tominaga et al. 2011; Nemoto et al. 2015). At the industrial and commercial scale, LA is available for production of organic chemicals by companies such as GFBiochemicals, which operates a 2-kt per year production facility in Caserta, Italy (Pileidis and Titirici 2016). Among products from the conversion of LA into useful chemicals, levulinate esters, γ-valerolactone, and alkanes of various molecular weights can be used as fuels and fuel additives, while γ-valerolactone can be used in the production of valuable monomers (Bozell et al. 2000; Pileidis and Titirici 2016).

In addition to catalytic reactions that produce commodity and functional chemicals from LA, research into the production of value-added chemicals such as pharmaceuticals, cosmetics, and biopolymers through biotechnological processes is also important (Jaremko and Yu 2011; Berezina and Yada 2016; Habe et al. 2015a, b). However, incomplete understanding of the pathways and enzymes involved in LA metabolism by microorganisms has limited the potential of LA-based biorefinery applications based on biotechnological processes.

**Fig. 1:**
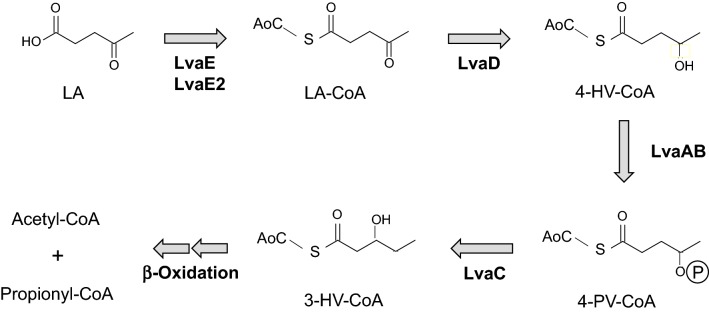
Proposed pathway and enzymes for the conversion of levulinic acid to 3-hydroxyvalelyl-CoA by *Pseudomonas* spp. *LA* levulinic acid, *LA-CoA* levulinyl-CoA, *4HV-CoA* 4-hydroxyvaleryl-CoA, *4-PV-CoA* 4-phosphovaleryl-CoA, *3-HV-CoA* 3-hydroxyvaleryl-CoA. The proposed pathway is quoted from Rand et al. (2017) with some modifications

Recently, Rand et al. clarified a seven-gene operon that is essential for assimilating LA into the β-oxidation pathway in *Pseudomonas putida* KT2440 (Rand et al. 2017). This excellent study reported that *lvaABCDE* genes were upregulated in the presence of LA and were involved in the conversion of LA to an intermediate of the β-oxidation pathway, 3-hydroxyvaleryl-coenzyme A (3HV-CoA; Fig. 1). Purified LvaE, which is homologous to acyl-CoA synthetases with a putative CoA-binding region and an AMP-binding site, catalyzed the ligation of CoA to LA to yield levulinyl-CoA (LA-CoA). LvaD (oxidoreductase) catalyzed the reduction of LA-CoA to 4-hydroxyvaleryl-CoA (4HV-CoA) with NADH or NADPH. Both *lvaA* and *lvaB*, and especially the former, which is homologous to the kinase superfamily and phosphotransferase family of enzymes, were required for growth on LA, and these two enzymes may catalyze the conversion of 4HV-CoA to 4-phospho-valeryl-CoA (4PV-CoA). LvaC (putative oxidoreductase) may be involved in the conversion of 4PV-CoA to 3HV-CoA via pentanoyl-CoA. These results suggest a unique catabolic pathway for LA, especially in that the isomerization of 4HV-CoA to 3HV-CoA proceeds through a phosphorylated intermediate, 4PV-CoA (Zhang et al. 2009; Harris et al. 2011; Rand et al. 2017).

Among KT2440 mutants with disrupted *lvaA*, *lvaB*, *lvaC*, and *lvaE* genes, Δ*lvaA*, Δ*lvaB*, and Δ*lvaC* strains were unable to grow on LA, indicating that these three genes were essential for LA catabolism in *P*. *putida*. By contrast, Δ*lvaE* could grow on LA, suggesting that LA is also activated by other CoA synthetases in *P*. *putida* (Rand et al. 2017). Besides *P*. *putida*, homologues to *lvaABCD* can be found in a variety of Alpha-, Beta-, and Gammaproteobacteria, but *lvaE* homologues were not investigated due to the high promiscuity of acyl-CoA ligases (Rand et al. 2017). Purified LvaE exhibited enzymatic activity toward C_4_ to C_6_ carboxylic acids, but low activity toward shorter or longer carboxylic acids, suggesting enzymatic specificity for fatty acids of moderate chain length. No information is available regarding the involvement of other acyl-CoA ligase homologues, especially those showing low homology to LvaE (e.g., less than 40%). Further studies on the diversity of the initial enzymes used in LA catabolism will be important for elucidating LA catabolism.

In this study, we isolated several genes of *Pseudomonas citronellolis* LA18T (Habe et al. 2015a, 2017; Inaba et al. 2018) that were upregulated in the presence of LA by comparative transcriptomic analysis using RNA sequencing (RNA-seq). As a CoA-synthetase with relatively low homology to LvaE was found within the gene cluster, we investigated its activity toward LA, and its structural features were compared with those of LvaE through model building.

## Materials and methods

All chemicals including LA were of the highest purity commercially available (98–100%; Sigma–Aldrich, Kanto Chemical, Wako Pure Chemical, Nacalai Tesque, Tokyo Chemical Industry). Levulinyl-CoA was synthesized by Tokyo Chemical Industry Co., Ltd. as a custom product.

### Bacterial strains, media, and cultivation

An LA-utilizing bacterium, *Pseudomonas citronellolis* LA18T (JCM 33429; Habe et al. 2015a, 2017; Inaba et al. 2018), was routinely cultivated on Luria–Bertani (LB) broth (composition: 10 g L^−1^ tryptone, 5 g L^−1^ yeast extract, and 10 g L^−1^ NaCl). For comparative transcriptome analysis, LA medium C (composition: 20 g L^−1^ reagent LA, 2 g L^−1^ K_2_HPO_4_, 5 g L^−1^ (NH_4_)_2_SO_4_, 0.25 g L^−1^ MgSO_4_, and 5 g L^−1^ yeast extract; pH 6.5) was used. As a reference, LA medium C containing 20 g L^−1^ pyruvic acid instead of 20 g L^−1^ LA (PA medium C) was used. Strain LA18T was pre-cultivated for 1 day in 3 mL LB broth in a test tube. Seed cultures (0.5 mL) were transferred to a 300-mL Erlenmeyer flask containing 50 mL of LA medium C or PA medium C, and cultured for 4 days at 30 °C on a rotary shaker (150 rpm). *Escherichia coli* strains JM109 and DH5α were routinely cultivated at 37 °C using LB broth, as described by Sambrook and Russell (2001). If necessary, ampicillin (Ap) was added to the medium at a final concentration of 50 µg mL^−1^. Cell growth was evaluated based on optical density (OD) measurement at 600 nm.

### RNA extraction and Illumina sequencing

Individual 2 mL samples of LA18T culture were collected on days 0, 2, and 3 during growth on LA (designated L0, L2, and L3, respectively) and on days 2 and 3 during growth on pyruvic acid (P2 and P3, respectively), for five samples in total. After centrifugation (15,300×*g*, 5 min, 4 °C), the resulting pellets were stored at − 80 °C until use. RNA was extracted from the pellets using the direct lysis protocol. Contaminating DNA was digested with DNase (RQ1 RNase-Free DNase, Promega, Fitchburg, WI, USA) and purified using the RNeasy Mini Kit (Qiagen, Venlo, Netherlands). The resultant total RNA was treated with a RiboMinus Transcriptome Isolation Kit for bacteria (Thermo Fisher Scientific, Waltham, MA, USA) to reduce rRNA levels, and then purified using an AMPure XP kit. Double-stranded cDNA libraries were prepared from the rRNA-depleted RNA samples using the NextUltraRNA library prep kit (New England Biolabs, Ipswich, MA, USA). Target-size cDNA (200–300 bp) was purified using the AMPure XP kit, followed by agarose gel electrophoresis. The size distribution and concentration of each purified cDNA sample was determined using a Bioanalyzer (Agilent 2100; Agilent Technologies, Santa Clara, CA, USA) and the Quant-iT PicoGreen dsDNA reagent and kit, respectively. An eight-picomolar cDNA sample was subjected to paired-end sequencing using a 300-cycle MiSeq reagent kit and MiSeq sequencer. RNA sequencing on the MiSeq platform provided 4,876,117 reads in total, corresponding to an average of 975,223 sequences per library (minimum, 699,410; maximum, 1,393,739).

### Quality checking and expression level analysis of RNA sequences

Paired-end Illumina reads from each of the five samples were checked for quality using FastQC (Andrews, S. [2010]; a quality control tool for high-throughput sequence data, available online at: http://www.bioinformatics.babraham.ac.uk/projects/fastqc). To ensure high sequence quality, the remaining sequencing adaptors, low-quality reads with a Phred score cutoff of 15 (leading and tailing sequences, Phred score > 20), and short reads of less than 100 bp were removed with the program Trimmomatic v0.30 using Illumina TruSeq 3 adapter sequences for adapter clipping (Bolger et al. 2014). Using genomic information for LA18T as a reference (DDBJ Sequence Read Archive accession codes BGPP01000001 and BGPP01000029; Inaba et al. 2018), paired-end RNA reads were mapped with the Bowtie2 program (Langmead and Salzberg 2012). After conversion of the output BAM files to BED files with the bamtobed program in BEDTools ver. 2.14.3 (Quinlan and Hall 2010), expression levels of the transcripts were evaluated by calculating transcripts per kilobase million (TPM) values using in-house scripts. The metatranscriptomic data obtained in this study have been deposited in the DDBJ Sequence Read Archive under accession code DRA007950.

### Cloning and expression of the acyl-CoA synthetase-encoding gene (*lvaE2*) in *E*. *coli*

Total DNA preparation, plasmid isolation, and restriction enzyme digestion were performed as described previously (Sambrook and Russell 2001). The DNA Ligation Kit version 2 (Takara, Japan) and a QIAEXII Gel Extraction Kit (Qiagen) were used according to the manufacturers’ instructions. Other commercially available enzymes and kits were used as described by their manufacturers. Expression vectors for PCLA_07r0387 (designated *lvaE2*) were constructed as follows: 1596-bp and 1566-bp DNA fragments containing the putative *lvaE2* gene were synthesized by Fasmac Co., Ltd., based on the LA18T genomic DNA sequence, to which *Nde*I and *Xba*I restriction sites were added at the 5′ and 3′ ends of the respective fragments. In the shorter DNA fragments (1566-bp), the 5′-terminal 30-bp region, corresponding to the N-terminal 10 amino acid sequence “MNFNLGIAAI”, was deleted from the longer DNA fragment, and the following methionine was used as a potential start codon for *lvaE2*. The longer and shorter DNA fragments were digested with two restriction enzymes then inserted into the corresponding sites in pCold™ IV DNA (Takara) to yield pCo7-387CoA-L and pCo7-387CoA-S, respectively.

*Escherichia coli JM109* or DH5α cells carrying pCo7-387CoA-L or pCo7-387CoA-S were pre-cultivated in 5 mL of LB medium with Ap at 37 °C for 18 h, and the seed cultures (1.5 mL) were transferred to 300-mL Erlenmeyer flasks containing 30 mL of LB medium with Ap. The flasks were incubated on a rotary shaker (200 rpm) at 37 °C until the OD_600_ reached 0.8–1.0, and then immediately cooled at 15 °C for 30 min. Following the addition of 0 to 1.0 mM isopropyl-β-d-thiogalactopyranoside (IPTG), the flasks were further incubated for 20 h at 15 °C. Then, the cells were harvested through centrifugation, washed twice with 50 mM sodium phosphate buffer (pH 7), and resuspended in 5 mL of the same buffer. The cell suspensions were sonicated and centrifuged at 20,400×*g* at 4 °C for 60 min, and the resultant supernatants were stored at − 80 °C until use as crude cell extracts. The protein concentration of each crude extract was determined using a protein assay kit (Bio-Rad Laboratories, Hercules, CA, USA). The production of LvaE2 was confirmed through SDS-PAGE with a 12.5% polyacrylamide gel. The gels were stained for protein detection with Coomassie Brilliant Blue Stain Solutions A and B (Nacalai Tesque).

### In vitro bioconversion of LA to levulinyl-CoA using crude enzymes

The enzymatic conversion of LA to LA-CoA with crude cell extracts containing LvaE2 was investigated by determining the time-dependent increase in the high-performance liquid chromatography (HPLC) peaks of LA-CoA. The reaction mixture (1500 μL total), which contained 50 mM sodium phosphate buffer (pH 7), 20 mM sodium levulinate (60 μL of 500 mM stock solution), 10 mM coenzyme A (HS-CoA; 15 μL of 1 M stock solution), 5 mM ATP (7.5 μL of 1 M stock solution), and approximately 300 μL of crude cell extract of *E*. *coli* JM109 or DH5α carrying pCo7-387CoA-L (0.4 μg mL^−1^), was incubated at 30 °C for 0 to 20 h. Samples for HPLC analysis were harvested at 0, 15, 30, 60, and 120 min and 20 h after beginning the incubation. As control experiments, reaction mixtures without HS-CoA or ATP were also tested. In addition, crude extracts of *E*. *coli* JM109 or DH5α cells carrying pCold™ IV DNA or pCo7-387CoA-S were used for comparison.

After centrifugation of the reaction mixtures, the supernatants were analyzed using HPLC with an LC-20AD HPLC pump (1.0 mL min^−1^ flow rate) and an SPD-20AV detector (Shimadzu) equipped with a TSK-GEL ODS-100 V column (150 × 4.6 mm, TOSOH)] to detect the peak from levulinyl-CoA. A mobile phase of 150 mM NaH_2_PO_4_ with 15% (v/v) methanol was used with this column. During analysis, the column temperature was maintained at 40 °C. The retention time of the reaction product was compared with that of synthesized LA-CoA. Co-chromatography experiments involving a mixed sample of the reaction product and synthesized LA-CoA were also performed.

### Phylogenetic tree construction and molecular model building

Multiple alignments were constructed with CLUSTAL W and phylogenetic analyses based on the neighbor-joining method were performed using MEGA7 software (Kumar et al. 2016). Molecular model building for LvaE2 from LA18T and LvaE from KT2440 was performed with an automated mode of SWISS-MODEL server (https://swissmodel.expasy.org/) using 1ult.1.A and 3r44.1 A as templates, respectively.

Briefly, suitable templates for the modeling analysis were identified based on BLAST and HHblits analyses of the amino acid sequences of the query proteins. Then, the templates showing high similarities scores were sorted according to the expected quality of the resulting models, as estimated primarily by Global Model Quality Estimation (GMQE) scores (For details, see the introduction of SWISS-MODEL: https://swissmodel.expasy.org/docs/help). Resultantly, top-ranked templates, i.e., 1ult.1.A and 3r44.1 A, were used for the 3D modeling. Structural analysis of the LvaE2 and LvaE models was performed using the PyMOL Molecular Graphics System (Version 1.4.1; Schrödinger, LLC).

## Results

### Comparative transcriptome analysis

Identification of genes involved in LA catabolism was carried out using a comparative RNA-seq technique with the transcriptomes of *P*. *citronellolis* LA18T grown on LA and pyruvic acid (Additional file 1: Figure S1). Comparative transcriptome analysis revealed some upregulated genes when LA18T cells were cultivated with LA. In LA18T cells, 571 genes in sample L3 were found to be upregulated by at least 2.0-fold compared with the P2 sample (L3/P2 ratio); the ten most upregulated genes are listed in Table 1. The two most strongly upregulated genes (PCLA_07r0385 and PCLA_07f0384) were located adjacent to each other in the genome and showed amino acid sequence homology with cation acetate symporters from *P*. *citronellolis* (99% identity) and *P*. *pseudoalcaligenes* (85% identity) and with 5-aminolevulinic acid dehydratases (ALADs; porphobilinogen synthase) from *P*. *citronellolis* (100% identity) and *P*. *putida* (93% identity), respectively.

**Table 1:** The ten most up-regulated genes of *Pseudomonas citronellolis* LA18T with levulinic acid

Scaffold	ORF	Product	Fold change^a^
scf_PCLA_07	PCLA_07r0385	Cation/acetate symporter	48.4
scf_PCLA_07	PCLA_07f0384	5-Aminolevulinic acid dehydratase	44.0
scf_PCLA_16	PCLA_16f0148	Putative metalloprotease	41.1
scf_PCLA_05	PCLA_05r0437	Putative structural toxin protein RtxA	30.8
scf_PCLA_05	PCLA_05f0439	TolC family type I secretion outer membrane protein	27.0
scf_PCLA_12	PCLA_12f0276	RNA polymerase sigma factor	21.6
scf_PCLA_15	PCLA_15f0186	Acetyl-CoA carboxylase	21.3
scf_PCLA_02	PCLA_02f0403	Adenylate kinase	18.5
scf_PCLA_01	PCLA_01f0750	NADH dehydrogenase	18.5
scf_PCLA_02	PCLA_02f0217	Putative membrane or periplasmic protein	15.0

^a^Expression of genes after 3-day cultivation with LA were compared to that after 2-day cultivation with pyruvic acid (L3/P2)

### Analysis of gene clusters containing genes upregulated in the presence of LA

**Fig. 2:**
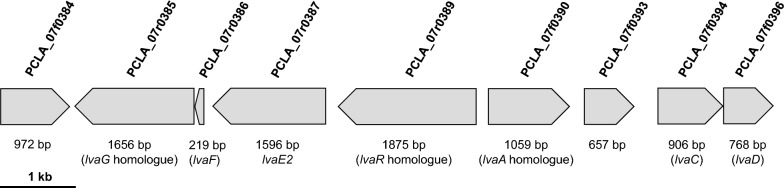
Organization of the gene cluster within the 11-kb DNA region of *Pseudomonas citronellolis* LA18T containing genes upregulated in the presence of levulinic acid. Blocks indicate the sizes, locations, and directions of transcription of the open reading frames (ORFs). ORF numbers and their sizes are noted beneath the blocks

To identify the open reading frames (ORFs) flanking the two most strongly upregulated genes, a genomic locus of approximately 11.1 kb containing PCLA_07r0385 and PCLA_07f0384 was re-analyzed in this study (PCLA_07f00384 to PCLA_07f0396; Fig. 2 and Table 2). The nucleotide sequence was deposited in the DDBJ, EMLB, and GenBank nucleotide sequence databases under accession number LC458675.

**Table 2:** Homology of annotated open reading frames in the 11.1-kb DNA region analyzed

ORF			Homologous protein			
	Length (aa)	Probable function	Protein	Source	Identity (aa %)	Accession no.
PCLA_07f0384	323	Porphobilinogen synthesis	5-Aminolevulinic acid dehydratase	*Pseudomonas citronellolis*	100	WP_074983815
			5-Aminolevulinic acid dehydratase (hemB)	*Pseudomonas putida* KT2440	93	PP_2913
PCLA_07r0385^a^	548	Organic acid transport	Cation/acetate symporter (ActP)	*Pseudomonas citronellolis*	99	WP_074983813
			Acetate permease (ActP-II)	*Pseudomonas putida* KT2440	64	PP_2797 (LvaG)
PCLA_07r0386^a^	72	Unknown	DFU485 domain-containing protein	*Pseudomonas citronellolis*	100	WP_074983811
			Hypothetical protein	*Pseudomonas putida* KT2440	31	PP_2796 (LvaF)
PCLA_07r0387^a^	531	Levulinyl-CoA synthetase	Long-chain fatty acid-CoA ligase	*Pseudomonas citronellolis*	99	WP_074983808
			Medium-chain fatty acid-CoA ligase	*Pseudomonas putida* KT2440	39 35	PP_0763, PP_2795 (LvaE)
PCLA_07r0389^a^	624	Levulinate catabolism operon regulator	Propionate catabolism operon regulatory protein (PrpR)	*Pseudomonas citronellolis*	100	WP_083426835
			sigma54-dependent sensory box protein	*Pseudomonas putida* KT2440	52	PP_2790 (LvaR)
PCLA_07f0390	352	Phosphotransferase family protein	Phosphotransferase family protein	*Pseudomonas citronellolis*	100	WP_074983804
			Aminoglycoside phospho transferase	*Pseudomonas putida* KT2440	59	PP_2791 (LvaA)
PCLA_07f0393	218	Phosphatase family protein	Histidine phosphatase family protein	*Pseudomonas citronellolis*	99	WP_074983800
			Alpha-ribazole-5′-phosphate phosphatase	*Pseudomonas putida* KT2440	32	PP_1680
PCLA_07f0394	301	Acyl-CoA dehydrogenase	Acyl-CoA dehydrogenase	*Pseudomonas citronellolis*	100	WP_074983798
			Acyl-CoA dehydrogenase	*Pseudomonas putida* KT2440	78	PP_2793 (LvaC)
PCLA_07f0396	255	Short-chain dehydrogenase reductase (SDR)	SDR family NAD(P)-dependent oxidoreductase	*Pseudomonas citronellolis*	100	WP_074983796
			SDR family oxidoreductase	*Pseudomonas putida* KT2440	67	PP_2794 (LvaD)

^a^ORFs are encoded in the complementary strand

I. PCLA_07f0384: ALAD. The deduced amino acid sequences shared the most homology with ALAD, which catalyzes the asymmetric condensation and cyclization of two 5-aminolevulinate molecules, the first common step in the biosynthesis of tetrapyrrole pigments such as porphyrin, chlorophyll, vitamin B12, siroheme, phycobilin, and cofactor F430 (Table 2). The product of PCLA_07f0384 contains a highly conserved metal-binding site for Zn^2+^, D-X-C-X-C-X-(Y/F)-X3-G-(H/Q)-C-G, but not an alternative cysteine-free site for Mg^2+^, D-X-A-L-D-X-(F/Y)-X3-G-(H/Q)-D-G (Jaffe 2016), suggesting an absolute Zn^2+^ requirement for the enzyme (data not shown).
II. PCLA_07r0385 to 07r0389: putative LA-assimilating gene cluster 1 (transporter and CoA ligase). We found a gene cluster of three ORFs encoding homologues of the acetate symporter ActP (PCLA_07r0385), fatty acid-CoA ligase (PCLA_07r0387), and regulatory protein (PCLA_07r0389) in the same direction of transcription. The polypeptide encoded by PCLA_07r0385, 07r0387, and 07r0389 showed 64%, 35%, and 52% identity, respectively, with LvaG, LvaE, and LvaR from *P*. *putida* KT2440 (Table 2). In addition, within the cluster, ORF PCLA_07r0386 (a homologue of a DUF485 domain-containing protein homologue; 72 amino acids) encoded a product with 35 to 39% identity with a hypothetical protein that clustered with the putative membrane protein ActP and inner membrane protein YjcH from *E*. *coli*. This protein is likely involved in organic acid transport along with PCLA_07r0385, as suggested for LvaF from *P*. *putida* (Rand et al. 2017).
III. PCLA_07f0390 to 07f0396: putative LA-assimilating gene cluster 2 (conversion of LA-CoA to 3HV-CoA). Four ORFs encoding possible LA-assimilating genes were located adjacent to the gene cluster of PCLA_07r0385 to 07r0389. The deduced amino acid sequence of PCLA_07f0390 shared 59% identity with the phosphotransferase family protein LvaA from *P*. *putida* KT2440. By contrast, the polypeptide encoded by PCLA_07f0393 showed 99% identity with a histidine phosphatase family protein from *P*. *citronellolis*. The deduced amino acid sequences of PCLA_07f0394 and 07f0396 showed 78% and 67% identities with the acyl-CoA dehydrogenase LvaC and short-chain dehydrogenase reductase LvaD, respectively, from *P*. *putida* KT2440.

### Expression of *lvaE2* (PCLA_07r0387) in *E*. *coli* cells

The gene PCLA_07r0387, encoding the fatty acid-CoA ligase homologue, was designated *lvaE2*. Expression vectors containing *lvaE2* were first constructed using the pET-26b(+) backbone with 6× His N-terminal fusion using the pET expression system with *E*. *coli* BL21 (DE3) cells. However, a band with a molecular mass of 59 kDa was observed in the precipitates but not in the supernatants. The same result was observed using the same expression system without His-tag fusion, i.e., native LvaE2 (data not shown).

Expression vectors containing *lvaE2* were then constructed using the pCold™ IV backbone, and IPTG-induced *E*. *coli* JM109 or DH5α cells carrying pCo7-387CoA-L were cultivated for the production of LvaE2. In both strains, JM109 and DH5α, the band at 59 kDa corresponding to LvaE2 was confirmed through SDS-PAGE from the supernatants as well as the precipitates (Additional file 1: Figure S2). By contrast, bands at 59 kDa were not observed in crude cell extracts of *E*. *coli* JM109 or DH5α cells containing not only the pCold™ IV vector (Additional file 1: Figure S1), but also pCo7-387CoA-S (data not shown).

### In vitro bioconversion of LA to levulinyl-CoA using crude enzymes

**Fig. 3:**
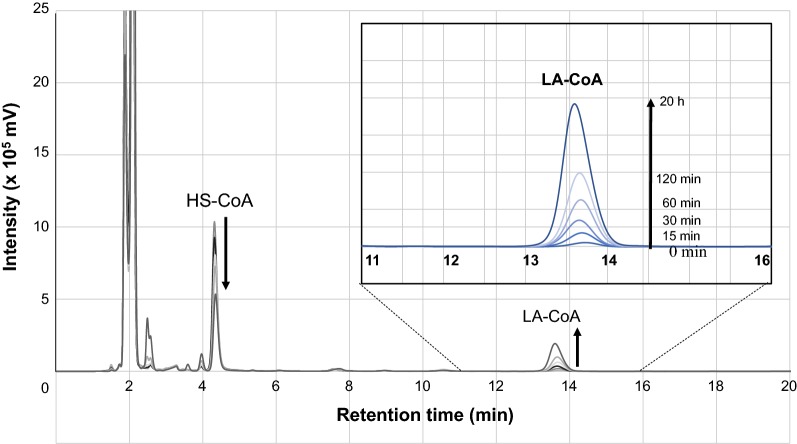
Formation of levulinyl-CoA (LA-CoA) from sodium levulinate, HS-CoA, and ATP in extracts of *E*. *coli* JM109 carrying the *lvaE2* gene (pCo7-387CoA-L) at 30 °C. HPLC analyses were performed using samples of the reaction mixture taken at 0, 15, 30, 60, and 120 min and 20 h after the addition of approximately 300 μL of crude cell extract (0.4 μg mL^−1^) into a total of 1500 mL reaction mixture. Up and down arrows indicate increasing and decreasing contents of LA-CoA and HS-CoA, respectively. Retention times of authentic HS-CoA and LA-CoA peaks were 4.3 min and 13.6 min, respectively

To investigate the levulinyl-CoA synthase activity of LvaE2, we monitored time-dependent LA-CoA production from LA by measuring the increase of HPLC peaks for LA-CoA. Using cell-free extracts of *E*. *coli* JM109 or DH5α expressing *lvaE2*, enzymatic activity was determined in the presence of sodium levulinate, HS-CoA, and ATP (Fig. 3). The HPLC peak area for LA-CoA (retention time: 13.6 min) increased from 0 min to 20 h, along with a decrease in the peak for HS-CoA (retention time: 4.3 min), whereas no peak for LA-CoA was observed in cell-free extracts of *E*. *coli* JM109 or DH5α cells carrying the pCold™ IV vector (control) or pCo7-387CoA-S. In addition, no peak for LA-CoA was detected in HPLC analyses when ATP or HS-CoA was excluded from the reaction mixture. The reaction also did not proceed with any samples from resuspended precipitates. These results indicate that LvaE2 (PCLA_07r0387 product) has enzymatic activity toward LA, and therefore acts as an LA-CoA synthase (LA-CoA ligase).

### Molecular modeling of LvaE2 from LA18T and LvaE from KT2440

**Fig. 4:**
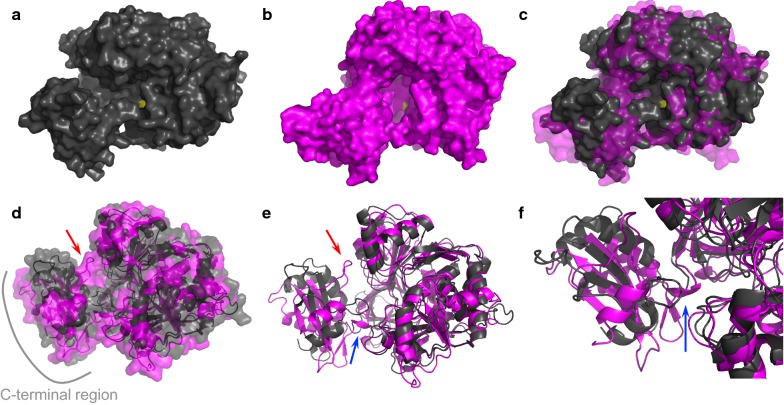
Modeled structures of LvaE2 from LA18T (black) and LvaE from KT2440 (magenta). Molecular surfaces (**a**–**d**) and structural models (**d**–**f**) were superimposed using PyMOL and the structures around the active site were compared. Red arrows indicate the predicted loop structure near the active site of LvaE. Blue arrows indicate the linker regions of LvaE and LvaE2. The location of the active site is shown in yellow (**a**–**c**)

Structural comparison was carried out through homology modeling of LvaE2 from LA18T and LvaE from KT2440. The two models have features similar to acyl-CoA synthetase from *Thermus thermophilus* (e.g., Hisanaga et al. 2004), including a small and globular C-terminal domain (Fig. 4a (LvaE2), b (LvaE), and d (superimposition), lower left part of the structure), a large and globular N-terminal domain (Fig. 4a, b, upper right part of the structure), and an active site located between these domains (yellow in Fig. 4a–c). Superimposition of the molecular surfaces of LvaE2 (black) and LvaE (magenta) revealed a predicted loop region in the C-terminal domain of the LvaE model, which is located in a tunnel region of LvaE2, as indicated by the red arrow in Fig. 4e. Although the overall structures of the C-terminal domains of the two models were relatively similar, the structures of the linker region between the C- and N-terminal domains (blue arrow in Fig. 4e, f) differed, resulting in a difference in the rotation angle of the C-terminal domains against the N-terminal domains. In LvaE, three amino acid residues (Ala437, Lys438, and Asp439) in the linker region form a short α-helix structure, whereas the equivalent three amino acid residues in LvaE2 (Arg428, Ile429, and Lys430) may form a loop structure (Fig. 4f).

## Discussion

The present study was undertaken to identify the genes involved in LA assimilation by *P*. *citronellolis* LA18T through RNA-seq-based comparative transcriptome analysis. The comparison of LA- and PA-grown LA18T cells allowed identification of the two most strongly upregulated genes (PCLA_07r0385 and PCLA_07f0384), which were highly homologous to cation acetate symporter and ALAD, respectively (Table 1). These two genes were located adjacent to each other, but with different transcriptional directions (Fig. 2).

In the DNA region containing PCLA_07r0385 and PCLA_07f0384, several other genes homologous to *lva* operon genes of KT2440 were identified (Fig. 2). In addition to an *lvaR* homologue (52% identity at the amino acid level; putative transcriptional regulator for LA assimilation), an LvaA homologue (59% identity) associated with the kinase superfamily and phosphotransferase family of enzymes was also observed (Fig. 2). In KT2440, LvaB, a hypothetical protein only 12 kDa in size, was demonstrated to interact with LvaA through a pulldown experiment, and both proteins LvaA and LvaB catalyzed the conversion of 4HV-CoA to 4PV-CoA (Rand et al. 2017). By contrast, in LA18T, no gene homologous to *lvaB* was found in the DNA region analyzed, but a gene homologous to histidine phosphatase family proteins was found adjacent to the *lvaA* homologue (PCLA_07f0393). This protein may be involved in the removal of a phosphate group from 4-PV-CoA in LA18T, although LvaC in KT2440 may be responsible for both phosphate group removal from 4-PV-CoA to produce enoyl-CoA and the subsequent hydration reaction producing 3-HV-CoA (Rand et al. 2017). In addition, the LvaC homologue in LA18T may participate in the reaction. Functional analysis of the histidine phosphatase homologue is necessary to clarify the difference between the mechanisms for transforming 4-PV-CoA into 3-HV-CoA in KT2440 and LA18T.

Among *lva* gene homologues in LA18T (Fig. 2), we demonstrated that the fatty acid-CoA ligase homologue, LvaE2, could catalyze the transformation of LA into LA-CoA through in vitro bioconversion experiments using crude enzymes from recombinant *E*. *coli*. In contrast to LvaE in KT2440, LvaE2 could not be produced in the soluble fraction using *E*. *coli* BL21 (DE3) cells with the pET expression system, although various cultivation conditions (e.g., IPTG concentration, temperature, and with or without the His-tag) were tested. The N-terminal His-tagged LvaE from KT2440 was successfully purified and investigated for its activity towards short- and medium-chain fatty acids, showing activity on C4–C6 carboxylic acids including LA and 4-hydroxyvaleric acid (Rand et al. 2017). In the CoA ligase activity assay, the amount of pyrophosphate released during the CoA ligase reaction was measured (absorbance at 360 nm). For this method, enzyme purification is needed because crude extracts contain some pyrophosphate and CoA ligases other than LvaE. By contrast, when investigating substrate specificity of LvaE2 in our bioconversion experiments, CoA derivatives of various carboxylic acids are needed as pure compounds for HPLC analysis, most of which are not commercially available. Although LvaE2 was hardly produced in the soluble fraction using *E*. *coli* cells with the pCold™ IV DNA system, increasing LvaE2 production through the use of other expression systems should be investigated for isolation of the enzyme.

Fatty acyl-CoA synthetases, required for conversion of long-chain fatty acids to their CoA thioesters, are defined based on the length of the aliphatic chain of their substrates as short-, medium-, and long-chain fatty acid CoA synthetases (Hisanaga et al. 2004). Enzymes in these classes use C2–C4, C4–C12, and C12–C22 fatty acids, respectively, as substrates. The functional role of the long-chain fatty acid CoA synthetase FadD from *E*. *coli* has been intensively studied, suggesting cooperative activity between FadD and FadL, the latter of which is an outer membrane fatty acid transport protein, that essentially renders the process of fatty acid transport unidirectional (Weimar et al. 2002). FadD activity was detected not only within the membrane but also in soluble fractions, suggesting that FadD moves between the bacterial cytosol and inner membrane to promote vectorial esterification of exogenous fatty acids (Mangroo and Gerber 1993). In addition, FadD was thought to be peripherally associated with the bacterial inner membrane (Mangroo and Gerber 1993). By contrast, LvaE from KT2440 is apparently classified as a medium-chain fatty acid CoA synthetase due to its utilization of C4–C6 carboxylic acids (Rand et al. 2017), and the recruitment process that brings medium-chain fatty acid CoA synthetases to the inner membrane remains unknown. The cooperative activity of LvaE and outer membrane proteins such as LvaG still remains to be investigated. Phylogenetic analysis revealed that LvaE2 from LA18T did not cluster with the long-chain fatty acid CoA synthetase FadD, but rather formed a cluster with the medium-chain fatty acid CoA synthetase LvaE. However, LvaE2 was not located on the same branch as LvaE and FadK (Additional file 1: Figure S3). The phylogenetic relationships among LvaE2, LvaE, and FadD suggest that LvaE2 may belong to medium-chain fatty acid CoA synthetases, but is phylogenetically distinct from LvaE in KT2440.

To clarify the differences between LvaE2 from LA18T and LvaE from KT2440, homology models of the two enzymes were compared. The overall structures of both the C- and N-terminal domains of the two models were relatively similar, whereas the structures of the linker region between the terminal domains (blue arrow in Fig. 4e, f) differed, causing a difference in the rotation angle of the C-terminal domains against the N-terminal domains. According to analysis of the structure of *Thermus thermophilus* acyl-CoA synthetase co-crystalized with myristoyl-AMP (Hisanaga et al. 2004), the length of the tunnel is hypothesized to determine substrate specificity, because myristoyl-AMP rests in the tunnel, which is well suited to accommodating its long hydrophobic tail. A similar mechanism has been proposed for the medium-chain specificity of the human homolog (Kochan et al. 2009). Considering these facts, the relatively rigid structure of the α-helix in the linker region of LvaE may determine the size of the tunnel region and cause strict substrate specificity for C4 to C6 fatty acids. Compared to LvaE, which has a predicted loop region at the entrance of the tunnel, no such loop region was located in the tunnel of LvaE2 (red arrow in Fig. 4e), suggesting that LvaE2 may exhibit substrate specificity toward fatty acids longer than those of LvaE. To investigate this prediction, a method for more efficient expression and purification of LvaE2 should be developed, but the structural differences between LvaE2 and LvaE may make it difficult to produce LvaE2 using *E*. *coli*.

In summary, a gene cluster that may be involved in LA catabolism was obtained from *P*. *citronellolis* LA18T using a comparative RNA-seq technique with LA18T cells grown on LA and pyruvic acid, providing the second example of LA catabolic genes following those of *P*. *putida* KT2440 (Rand et al. 2017). Among the genes identified, the initial enzyme for LA catabolism, levulinyl-CoA synthase LvaE2, which differs phylogenetically from LvaE in KT2440, was produced in *E*. *coli* cells and was confirmed to have activity toward LA. Among the genes encoding subsequent enzymatic steps, some differences were found between LA18T and KT2440 *lva* gene clusters; in particular, LvaB is not found within the gene cluster in LA18T, and instead, a gene encoding a phosphatase-family protein is located in the cluster. Further functional analysis studies of LA18T *lva* genes are necessary to elucidate the detailed mechanisms of LA metabolism.

## Additional file

**Additional file 1: Figure S1.** Growth of *Pseudomonas citronellolis* LA18T on levulinic acid and pyruvic acid. OD_600_ values were measured. Symbols: black circle, levulinic acid; white circle, pyruvic acid. The vertical bar indicates the variation between two independent experiments. **Figure S2.** SDS-PAGE analysis of the soluble and insoluble fractions from recombinant *E*. *coli* cells carrying pCo7-387CoA-L and pCold™ IV. Lanes: M, molecular mass marker; 1 and 2, insoluble and soluble fractions from JM109 cells carrying pCo7-387CoA-L; 3, soluble fraction from JM109 cells carrying pCold™ IV; 4 and 5, insoluble and soluble fractions from DH5α cells carrying pCo7-387CoA-L. The crude extracts were applied to SDS-PAGE at a concentration of 0.5 μg/mL. **Figure S3.** Unrooted phylogenetic tree showing the positions of LvaE2 from *Pseudomonas citronellolis* (closed circle), LvaE from *Pseudomonas putida* (open circle), FadD from *E*. *coli* (closed triangle), and some other fatty acyl-CoA synthetases (FadK, open triangle; PrpE, closed square) based on multiple alignment of related proteins. The amino acid sequences used for phylogenetic analysis were collected through BLAST searches (https://blast.ncbi.nlm.nih.gov/Blast.cgi) using LvaE2, LvaE, *E*. *coli* FadD (long-chain fatty acid**-**CoA ligase; YP_006120162.1), *E*. *coli* FadK (short chain acyl-CoA synthetase; NP_416216.5), and *E*. *coli* PrpE (propionyl-CoA synthetase; NP_414869.1) as queries against the RefSeq database. As a result, 55, 79, 99, 55, and 62 sequences with similarity to LvaE2, LvaE, FadD, FadK, and PrpE, respectively, were obtained. The major constituents are: Group I (including FadD), long-chain fatty acid**-**CoA ligases; Group II, AMP-binding proteins; Group III (including LvaE2), long-chain fatty acid**-**CoA ligases; Group IV (including LvaE), acyl-CoA synthetases; Group V (FadK), short-chain acyl-CoA synthetases and cyclohexane carboxylate CoA ligases; and Group VI (PrpE), propionyl-CoA synthetases and acetate CoA ligases.

## Data Availability

*Pseudomonas citronellolis* LA18T has been deposited in RIKEN BRC under accession number JCM 33429. The nucleotide sequence was deposited in the DDBJ, EMLB, and GenBank nucleotide sequence databases under accession number LC458675.
