# Supplementary material for: Identification and characterization of levulinyl-CoA synthetase from Pseudomonas citronellolis, which differs phylogenetically from LvaE of Pseudomonas putida
Source: AMB Express. 2019 Aug 13;9:127. doi: 10.1186/s13568-019-0853-y (PMC6692424; doi:10.1186/s13568-019-0853-y)
Supplement: Supplementary file 1 — Additional file 1: Figure S1. Growth of Pseudomonas citronellolis LA18T on levulinic acid and pyruvic acid. OD600 values were measured. Symbols: black circle, levulinic acid; white circle, pyruvic acid. The vertical bar indicates the variation between two independent experiments. Figure S2. SDS-PAGE analysis of the soluble and insoluble fractions from recombinant E. coli cells carrying pCo7-387CoA-L and pCold™ IV. Lanes: M, molecular mass marker; 1 and 2, insoluble and soluble fractions from JM109 cells carrying pCo7-387CoA-L; 3, soluble fraction from JM109 cells carrying pCold™ IV; 4 and 5, insoluble and soluble fractions from DH5α cells carrying pCo7-387CoA-L. The crude extracts were applied to SDS-PAGE at a concentration of 0.5 μg/mL. Figure S3. Unrooted phylogenetic tree showing the positions of LvaE2 from Pseudomonas citronellolis (closed circle), LvaE from Pseudomonas putida (open circle), FadD from E. coli (closed triangle), and some other fatty acyl-CoA synthetases (FadK, open triangle; PrpE, closed square) based on multiple alignment of related proteins. The amino acid sequences used for phylogenetic analysis were collected through BLAST searches (https://blast.ncbi.nlm.nih.gov/Blast.cgi) using LvaE2, LvaE, E. coli FadD (long-chain fatty acid-CoA ligase; YP_006120162.1), E. coli FadK (short chain acyl-CoA synthetase; NP_416216.5), and E. coli PrpE (propionyl-CoA synthetase; NP_414869.1) as queries against the RefSeq database. As a result, 55, 79, 99, 55, and 62 sequences with similarity to LvaE2, LvaE, FadD, FadK, and PrpE, respectively, were obtained. The major constituents are: Group I (including FadD), long-chain fatty acid-CoA ligases; Group II, AMP-binding proteins; Group III (including LvaE2), long-chain fatty acid-CoA ligases; Group IV (including LvaE), acyl-CoA synthetases; Group V (FadK), short-chain acyl-CoA synthetases and cyclohexane carboxylate CoA ligases; and Group VI (PrpE), propionyl-CoA synthetases and acetate CoA ligases. [file 13568_2019_853_MOESM1_ESM.doc]

JOURNAL NAME: AMB express

Identification and characterization of levulinyl-CoA synthetase from *Pseudomonas citronellolis*, which differs phylogenetically from LvaE

of *Pseudomonas putida*

Hiroshi Habe ^1*^ • Hideaki Koike ^2^ • Yuya Sato ^1^ • Yousuke Iimura ^1^ • Tomoyuki Hori ^1^ • Manabu Kanno ^2^ • Nobutada Kimura ^2^ • Kohtaro Kirimura^3^

^1^Environmental Management Research Institute, National Institute of Advanced Industrial Science and Technology (AIST), Tsukuba West, 16-1 Onogawa, Tsukuba, Ibaraki 305-8569, Japan

^2^Bioproduction Research Institute, AIST, Tsukuba Central 6-10, 1-1-1 Higashi, Tsukuba, Ibaraki 305-8566, Japan

^3^Department of Applied Chemistry, Faculty of Science and Engineering, Waseda University, Tokyo 169-8555, Japan

^*^Corresponding author. Phone: +81-29-861-6247; Fax: +81-29-861-4674; E-mail: hiroshi.habe@aist.go.jp

**Figure S1.** Growth of *Pseudomonas* *citronellolis* LA18T on levulinic acid and pyruvic acid. OD_600_ values were measured. Symbols: black circle, levulinic acid; white circle, pyruvic acid. The vertical bar indicates the variation between two independent experiments.

**Figure S2.** SDS-PAGE analysis of the soluble and insoluble fractions from recombinant *E*. *coli* cells carrying pCo7-387CoA-L and pCold^TM^ IV. Lanes: M, molecular mass marker; 1 and 2, insoluble and soluble fractions from JM109 cells carrying pCo7-387CoA-L; 3, soluble fraction from JM109 cells carrying pCold^TM^ IV; 4 and 5, insoluble and soluble fractions from DH5α cells carrying pCo7-387CoA-L. The crude extracts were applied to SDS-PAGE at a concentration of 0.5 μg/mL.

**Figure S3.** Unrooted phylogenetic tree showing the positions of LvaE2 from *Pseudomonas citronellolis* (closed circle), LvaE from *Pseudomonas putida* (open circle), FadD from *E*. *coli* (closed triangle), and some other fatty acyl-CoA synthetases (FadK, open triangle; PrpE, closed square) based on multiple alignment of related proteins. The amino acid sequences used for phylogenetic analysis were collected through BLAST searches (https://blast.ncbi.nlm.nih.gov/Blast.cgi) using LvaE2, LvaE, *E*. *coli* FadD (long-chain fatty acid**-**CoA ligase; YP_006120162.1), *E*. *coli* FadK (short chain acyl-CoA synthetase; NP_416216.5), and *E*. *coli* PrpE (propionyl-CoA synthetase; NP_414869.1) as queries against the RefSeq database. As a result, 55, 79, 99, 55, and 62 sequences with similarity to LvaE2, LvaE, FadD, FadK, and PrpE, respectively, were obtained. The major constituents are: Group I (including FadD), long-chain fatty acid**-**CoA ligases; Group II, AMP-binding proteins; Group III (including LvaE2), long-chain fatty acid**-**CoA ligases; Group IV (including LvaE), acyl-CoA synthetases; Group V (FadK), short-chain acyl-CoA synthetases and cyclohexane carboxylate CoA ligases; and Group VI (PrpE), propionyl-CoA synthetases and acetate CoA ligases.
